# ETHE1 Accelerates Triple-Negative Breast Cancer Metastasis by Activating GCN2/eIF2α/ATF4 Signaling

**DOI:** 10.3390/ijms241914566

**Published:** 2023-09-26

**Authors:** Shao-Ying Yang, Li Liao, Shu-Yuan Hu, Ling Deng, Lisa Andriani, Tai-Mei Zhang, Yin-Ling Zhang, Xiao-Yan Ma, Fang-Lin Zhang, Ying-Ying Liu, Da-Qiang Li

**Affiliations:** 1 Fudan University Shanghai Cancer Center and Institutes of Biomedical Sciences, Fudan University, Shanghai 200032, China; 19211510017@fudan.edu.cn (S.-Y.Y.); 16211220054@fudan.edu.cn (L.L.); 18111510026@fudan.edu.cn (S.-Y.H.); 20111510058@fudan.edu.cn (L.D.); 17111510026@fudan.edu.cn (T.-M.Z.); 2 Cancer Institute, Shanghai Medical College, Fudan University, Shanghai 200032, China; 23111230017@fudan.edu.cn (Y.-L.Z.); zhangfanglin555@sina.com (F.-L.Z.); 3 Department of Oncology, Shanghai Medical College, Fudan University, Shanghai 200032, China; 4 Department of Breast Surgery, Shanghai Medical College, Fudan University, Shanghai 200032, China; 20111230078@fudan.edu.cn (L.A.); xyma20@fudan.edu.cn (X.-Y.M.); 5 Shanghai Key Laboratory of Breast Cancer, Shanghai Medical College, Fudan University, Shanghai 200032, China; 6 Shanghai Key Laboratory of Radiation Oncology, Shanghai Medical College, Fudan University, Shanghai 200032, China

**Keywords:** triple-negative breast cancer, ethylmalonic encephalopathy protein 1, cancer invasion and metastasis, integrated stress response

## Abstract

Triple-negative breast cancer (TNBC) is the most fatal subtype of breast cancer; however, effective treatment strategies for TNBC are lacking. Therefore, it is important to explore the mechanism of TNBC metastasis and identify its therapeutic targets. Dysregulation of ETHE1 leads to ethylmalonic encephalopathy in humans; however, the role of ETHE1 in TNBC remains elusive. Stable cell lines with ETHE1 overexpression or knockdown were constructed to explore the biological functions of ETHE1 during TNBC progression in vitro and in vivo. Mass spectrometry was used to analyze the molecular mechanism through which ETHE1 functions in TNBC progression. ETHE1 had no impact on TNBC cell proliferation and xenograft tumor growth but promoted TNBC cell migration and invasion in vitro and lung metastasis in vivo. The effect of ETHE1 on TNBC cell migratory potential was independent of its enzymatic activity. Mechanistic investigations revealed that ETHE1 interacted with eIF2α and enhanced its phosphorylation by promoting the interaction between eIF2α and GCN2. Phosphorylated eIF2α in turn upregulated the expression of ATF4, a transcriptional activator of genes involved in cell migration and tumor metastasis. Notably, inhibition of eIF2α phosphorylation through ISRIB or ATF4 knockdown partially abolished the tumor-promoting effect of ETHE1 overexpression. ETHE1 has a functional and mechanistic role in TNBC metastasis and offers a new therapeutic strategy for targeting ETHE1-propelled TNBC using ISRIB.

## 1. Introduction

Breast cancer is not only a common malignant tumor but also the second leading cause of tumor-related deaths in females [1]. As the most aggressive subtype of breast cancer, triple-negative breast cancer (TNBC) lacks the expression of estrogen and progesterone receptors as well as human epidermal growth factor receptor 2 [2]. Additionally, TNBC is considered as one of the most fatal malignancies because of its high risk of recurrence, metastasis, poor clinical outcome, and lack of available therapeutic targets [2,3]. Therefore, there is an urgent need to identify new targets that drive TNBC progression.

The human metallo β-lactamase fold (hMBLf) protein family includes 18 zinc- and iron-dependent enzymes that catalyze a series of biochemical reactions, including hydrolysis and redox reactions [4]. Phylogenetic analysis divides hMBLf proteins into three subfamilies: glyoxalase II, the DNA/RNA interacting subfamily, and a subfamily with more diversified functions [4]. Ethylmalonic encephalopathy protein 1 (ETHE1) belongs to the glyoxalase II subfamily and is located in the nucleus, mitochondrial matrix, and cytosol [5,6,7]. ETHE1 has sulfur dioxygenase activity and can catalyze the dioxygenation of glutathione persulfide to glutathione and sulfite to facilitate hydrogen sulfide (H_2_S) catabolism in the mitochondrial matrix [8,9]. This process is essential for mitochondrial metabolic homeostasis [10,11]. Loss of or mutations in ETHE1 leads to ethylmalonic encephalopathy in humans, characterized by brain damage, lactic acidemia, and even death [6,12,13]. Despite its pivotal role in ethylmalonic encephalopathy, whether dysregulation of ETHE1 is implicated in human cancer is poorly understood. Emerging evidence shows that ETHE1 is upregulated in colorectal adenocarcinoma compared with benign colonic epithelium and it may also contribute to colorectal tumorgenicity [14,15]. Additionally, ETHE1 forms a biomarker panel with H-Ras and p53 to diagnose early-stage lung cancer [16]. Nevertheless, the biological function and related molecular mechanism of ETHE1 in TNBC progression remain largely unexplored.

In this study, we first demonstrated that ETHE1 plays a key role in TNBC metastasis without affecting TNBC cell and tumor growth. Notably, the effects of ETHE1 on TNBC cells were independent of its enzymatic activity. Mechanistic investigations revealed that ETHE1 enhanced kinase general control nonderepressible 2 (GCN2)-mediated phosphorylation of eukaryotic translation initiation factor 2 subunit alpha (eIF2α), which in turn promoted the expression of activating transcription factor 4 (ATF4). Moreover, pharmacological inhibition of eIF2α phosphorylation using integrated stress response inhibitor (ISRIB) or depletion of ATF4 partially abolished the tumor-promoting effects of ETHE1 on TNBC metastasis both in vitro and in mice. In conclusion, these findings demonstrate that ETHE1 can serve as a novel therapeutic target for TNBC.

## 2. Results

### 2.1. ETHE1 Is Upregulated in TNBC Tissues and Its High Expression Predicts Poor TNBC Prognosis

**Figure 1:**
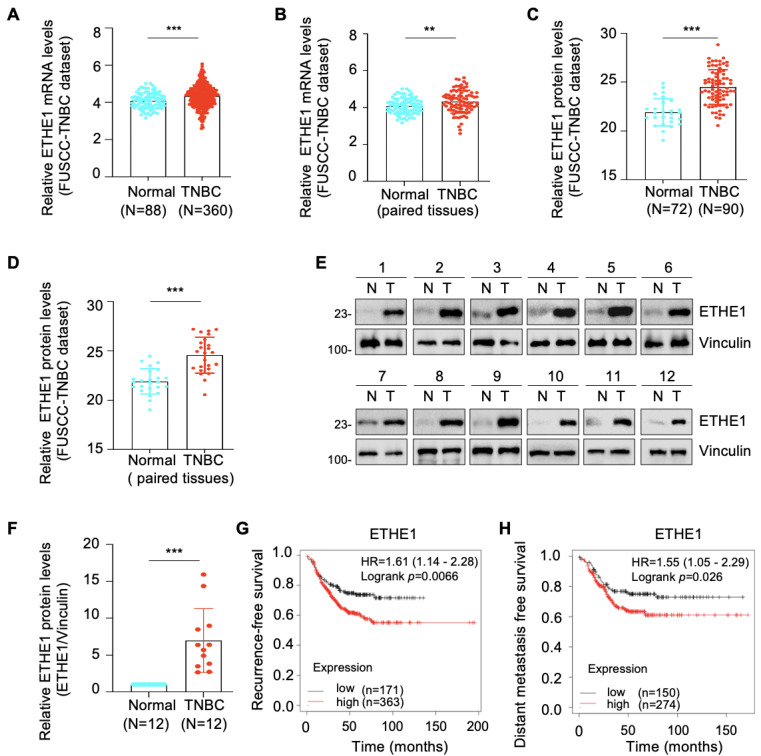
ETHE1 is highly expressed in TNBC tissues and predicts poor TNBC prognosis. (**A**,**B**) Analysis of ETHE1 mRNA levels in transcriptomic datasets from the FUSCC-TNBC cohort. (**C**,**D**) Analysis of ETHE1 protein levels in proteomic datasets from the FUSCC-TNBC cohort. (**E**,**F**) Immunoblotting analysis of ETHE1 protein levels in 12 pairs of TNBC tissues and adjacent non-cancerous samples (**E**) and quantitative results of the two groups are shown in (**F**). (**G**,**H**) Kaplan–Meier analysis of RFS and DMFS of patients with TNBC using a breast cancer transcriptomic dataset from the Kaplan–Meier plotter database. ** p < 0.01; *** p < 0.001.

Transcriptomic [17] and proteomic [18] datasets from the FUSCC-TNBC cohort were used to analyze the expression profile of ETHE1 in TNBC. Notably, the mRNA (Figure 1A,B) and protein (Figure 1C,D) expression levels of ETHE1 were significantly elevated in TNBC tissues compared with noncancerous breast tissues. We collected an additional 12 pairs of TNBC tissues and adjacent noncancerous samples and measured the ETHE1 protein levels via immunoblotting. The above results further suggest that ETHE1 expression is higher in TNBC tissues than in their normal counterparts (Figure 1E,F). Moreover, analysis of a breast cancer transcriptome dataset from the Kaplan–Meier plotter database revealed that patients with ETHE1 overexpression had worse recurrence-free survival and distant metastasis-free survival statuses (Figure 1G,H). Together, the above results indicate that ETHE1 is significantly overexpressed in TNBC tissues and signals poor clinical outcomes.

### 2.2. ETHE1 Does Not Affect TNBC Cell Proliferation or Xenograft Tumor Growth

**Figure 2:**
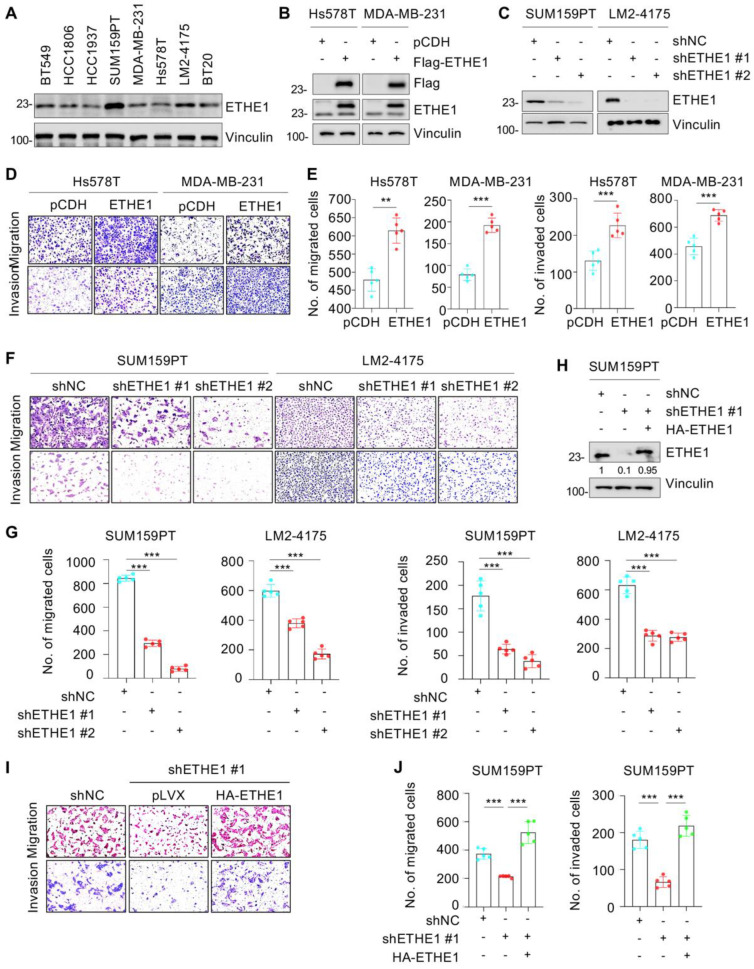
ETHE1 promotes the migratory and invasive abilities of TNBC cells. (**A**) Detection of ETHE1 protein levels in eight TNBC cell lines via immunoblotting. (**B**) Detection of ETHE1 expression status in ETHE1-overexpressing cells via immunoblotting. (**C**) Detection of ETHE1 knockdown efficiency in ETHE1-knockdown cells via immunoblotting. (**D**–**G**) Trans-well assays were used to detect the effects of ETHE1 overexpression (**D**,**E**) or knockdown (**F**,**G**) on the migratory and invasive abilities of TNBC cells. Typical images (**D**,**F**) and quantitative results (**E**,**G**) are shown. (**H**) Detection of the expression status of ETHE1 in SUM159PT cells expressing shNC, shETHE1 #1 alone, or shETHE1 #1 in combination with HA-ETHE1. (**I**,**J**) SUM159PT cells expressing shNC, shETHE1 #1 alone, or shETHE1 #1 in combination with HA-ETHE1 were performed to Trans-well assays. Typical images (**I**) and quantitative results (**J**) are shown. ** p < 0.01; *** p < 0.001.

To delineate the role of ETHE1 in TNBC cells, we determined the endogenous levels of ETHE1 in eight representative TNBC cell lines via immunoblotting. Figure 2A shows that the levels of ETHE1 protein were higher in SUM159PT and LM2-4175 cell lines than in the other six cell lines. Next, we overexpressed Flag-ETHE1 in Hs578T and MDA-MB-231 cell lines (Figure 2B) and depleted endogenous ETHE1 in SUM159PT and LM2-4175 cell lines using two separate shRNAs targeting ETHE1 (shETHE1 #1 and #2) (Figure 2C). The overexpression or knockdown status in the resultant cells was confirmed using immunoblotting analysis. CCK-8 (Supplementary Figure S1A) and colony formation assays (Supplementary Figure S1B,C) showed that ETHE1 overexpression had no effect on the growth and colony formation capability of Hs578T and MDA-MB-231 cells in vitro. A consistent phenomenon was also observed upon ETHE1 knockdown (Supplementary Figure S1D–F). To assess whether ETHE1 affects tumorigenesis in vivo, MDA-MB-231 cells stably overexpressing vector or ETHE1 were injected into the mammary fat pad of BALB/c female nude mice (5-week-old). As shown in Supplementary Figure S1G and H, no prominent differences were observed in the size and weight of xenograft tumors between ETHE1-overexpressing and empty-vector-expressing groups. These results collectively suggest that ETHE1 has no effect on TNBC cell proliferation and xenograft tumor growth.

### 2.3. ETHE1 Promotes TNBC Cell Migration and Invasion, and the Noted Effects Are Independent of Its Enzymic Activity

We further investigated whether ETHE1 contributes to TNBC cell metastasis. Trans-well migration and invasion experiments suggested that ETHE1 overexpression significantly boosted migratory and invasive rates in Hs578T and MDA-MB-231 cells (Figure 2D,E). Consistently, ETHE1 knockdown significantly inhibited the migratory and invasive capabilities of SUM159PT and LM2-4175 cells (Figure 2F,G). To further confirm these results, we reintroduced HA-ETHE1 into ETHE1-depleted SUM159PT cells (Figure 2H) and found that restoration of ETHE1 expression in ETHE1-depleted cells reversed the lowered migratory and invasive rates of SUM159PT cells caused by ETHE1 knockdown (Figure 2I,J). Generally, these results demonstrate that ETHE1 boosted the migratory and invasive potentials of TNBC cells.

It has been reported that four mutants of ETHE1—T152I [19], R163W [20,21], D196N [19], and C247S [22] (Figure S2A)—can affect its enzymatic activity. To verify whether ETHE1-mediated TNBC cell migration and invasion depend on its enzymatic activity, we overexpressed the wild-type and four mutants of ETHE1 in Hs578T and MDA-MB-231 cells via lentiviral infections and performed Trans-well migration assays (Figure S2B). The results showed that the expression of all four ETHE1 mutations did not significantly affect its capacity to promote TNBC cell migration compared with its wild-type counterpart (Figure S2C–E). The foregoing data suggest that the role of ETHE1 in promoting TNBC cell migration is independent of its enzymatic activity.

### 2.4. ETHE1 Interacts with eIF2α

**Figure 3:**
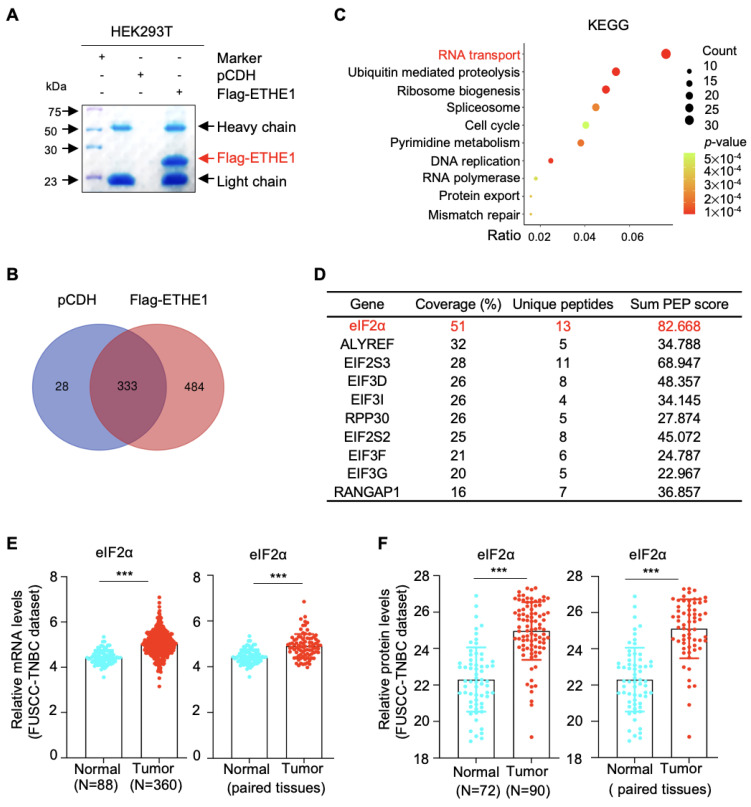
Identification of ETHE1-interacting proteins using LC-MS/MS. (**A**) HEK293T cells overexpressing vector or Flag-ETHE1 were analyzed using IP assays with anti-Flag beads. (**B**) Identified proteins with three or more unique peptides were subjected to Venn analysis. (**C**) KEGG pathway analysis was performed on the ETHE1-interacting proteins. (**D**) The top 10 proteins interacted with ETHE1 in the RNA transport pathway. (**E**) Analysis of eIF2α mRNA levels in the transcriptomic datasets from the FUSCC-TNBC cohort. (**F**) Analysis of eIF2α protein levels in the proteomic datasets from the FUSCC-TNBC cohort. *** p < 0.001.

To identify the molecular mechanisms of ETHE1 that promote TNBC metastasis, we examined the potential interacting partners of ETHE1 in HEK293T cells overexpressing vector or Flag-ETHE1. Total cellular lysates were analyzed using IP assays with anti-Flag beads. After separation and staining, the gel was subjected to LC-MS/MS analysis (Figure 3A). Proteomic analysis revealed that 484 proteins showed potential interactions with ETHE1 (Figure 3B). To explore the signaling pathways associated with ETHE1-interacting proteins, we performed KEGG analysis and found that the RNA transport signaling pathway ranked first (Figure 3C). Proteins that participate in RNA transport were subsequently sorted based on coverage percentage (Figure 3D). We selected the eIF2α protein for further validation as it ranked first. Further, analysis of FUSCC-TNBC transcriptomic [17] and proteomic [18] datasets found that eIF2α was upregulated in TNBC tissues at the transcriptional and protein levels (Figure 3E,F). The above results suggest that eIF2α might be a new binding partner of ETHE1.

**Figure 4:**
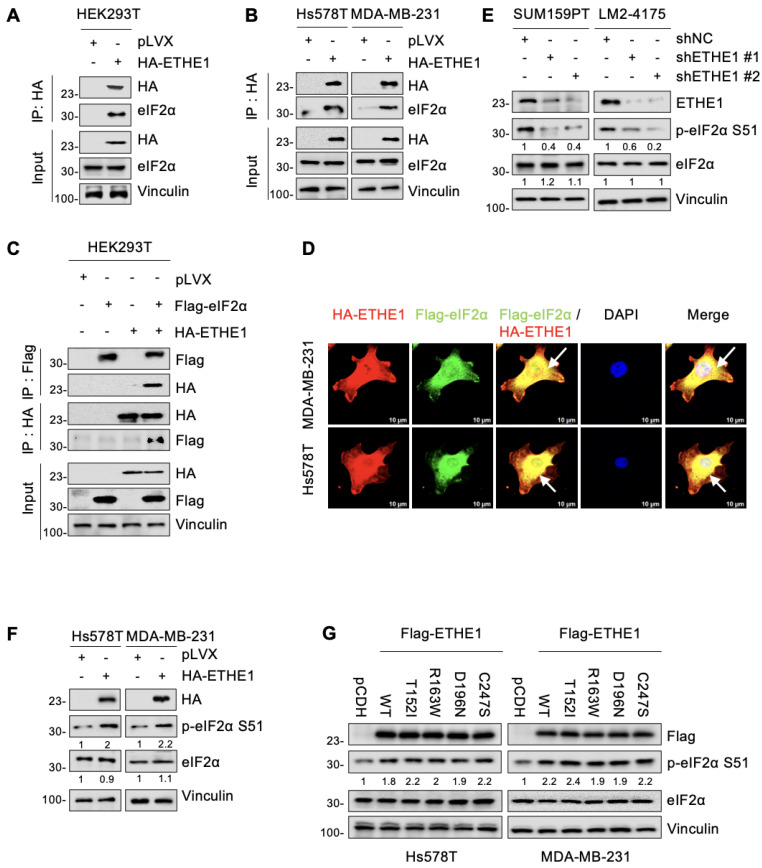
ETHE1 interacts with eIF2α and enhances its phosphorylation. (**A**,**B**) HEK293T and TNBC cells overexpressing vector or HA-ETHE1 were analyzed using IP assays with anti-HA beads and then detected using immunoblotting. (**C**) HEK293T cells were transiently transfected with the corresponding plasmids for IP experiments as well as immunoblotting analysis to verify the interaction between ETHE1 and eIF2α. (**D**) Immunofluorescent staining was used to detect the colocalization of ETHE1 with eIF2α in TNBC cells overexpressing HA-ETHE1 (red) and Flag-eIF2α (green) in combination, and nuclei were counterstained with DAPI (blue). Typical colocalizations between HA-ETHE1 and Flag-EIF2S1 are indicated with the white arrows. (**E**,**F**) Immunoblotting experiments were performed to detect the total protein and phosphorylation expression status of eIF2α in ETHE1-knockdown (**E**) and ETHE1-overexpressing cells (**F**), respectively. (**G**) Immunoblotting experiments were performed to detect the total protein and phosphorylation expression status of eIF2α in cells expressing wild-type and mutant ETHE1.

To investigate whether ETHE1 interacts with eIF2α, vector or ETHE1 were transiently or stably overexpressed in HEK293T, Hs578T, and MDA-MB-231 cells and subjected to IP assays. As shown in Figure 4A,B, eIF2α indeed interacted with ETHE1 in those cells. Additionally, ETHE1 and eIF2α carrying different tags showed reciprocal interactions on co-expression (Figure 4C). Immunofluorescent staining also indicated that ETHE1 and eIF2α co-localized in Hs578T and MDA-MB-231 cells (Figure 4D). Together, these results demonstrated that ETHE1 interacted with eIF2α.

### 2.5. ETHE1 Enhances eIF2α Phosphorylation

We next determined whether there is a regulatory relationship between ETHE1 and eIF2α. Notably, both knockdown of endogenous ETHE1 in SUM159PT and LM2-4175 cells and ectopic expression of ETHE1 in Hs578T and MDA-MB-231 cells did not significantly affect the total eIF2α protein levels (Figure 4E,F). As eIF2α phosphorylation at serine 51 (p-eIF2α S51) is required for cell migration [23], we examined whether ETHE1 affects eIF2α phosphorylation. Immunoblotting experiments revealed that ETHE1 knockdown downregulated eIF2α phosphorylation while ETHE1 overexpression upregulated eIF2α phosphorylation (Figure 4E,F). Intriguingly, this phenomenon was also observed in cells expressing mutant ETHE1, and there were no differences in the levels of phosphorylated eIF2α between cells expressing WT and mutant ETHE1 (Figure 4G). This result is consistent with the observation that ETHE1 mutations did not affect ETHE1-mediated migration of TNBC cells. Overall, ETHE1 affects the phosphorylation levels of eIF2α rather than its total protein levels.

### 2.6. ETHE1 Enhances eIF2α Phosphorylation by Promoting the Interaction between eIF2α and GCN2

**Figure 5:**
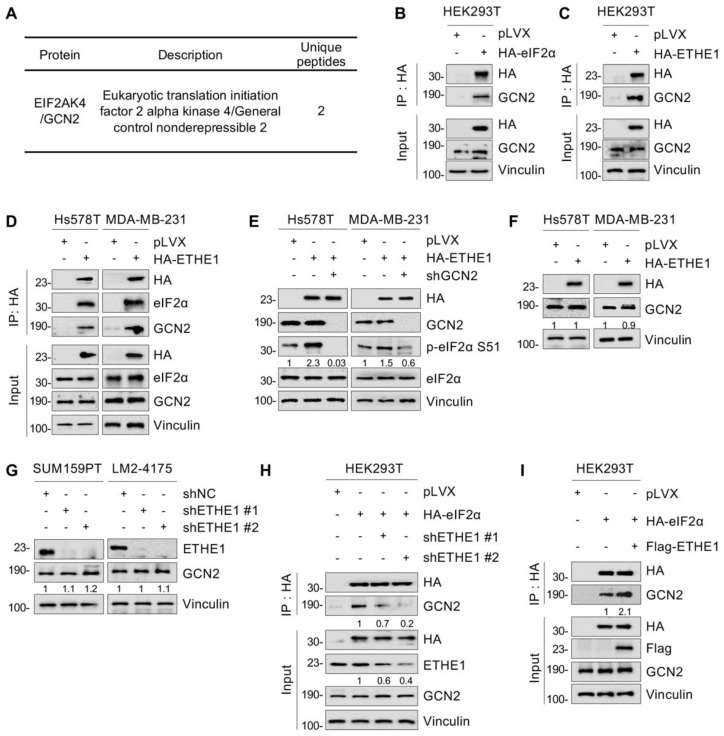
ETHE1 enhances eIF2α phosphorylation by promoting the interaction between eIF2α and GCN2. (**A**) Identification of GCN2 as a potential ETHE1-interacting protein using LC-MS/MS. (**B**,**C**) HEK293T cells were transiently transfected with the corresponding plasmids for IP experiments and immunoblotting analysis to verify the interactions between eIF2α and GCN2 and between ETHE1 and GCN2. (**D**) TNBC cells overexpressing vector or HA-ETHE1 were analyzed using IP assays with anti-HA beads and then detected via immunoblotting. (**E**) Immunoblotting experiments were performed to detect the total protein and phosphorylation statuses of eIF2α in ETHE1-overexpressing cells infected with or without shGCN2 lentiviruses. (**F**,**G**) Immunoblotting experiments were performed to detect the expression of GCN2 in ETHE1-overexpressing (**F**) and ETHE1-knockdown cells (**G**), respectively. (**H**,**I**) HEK293T HA-eIF2α cells were knockdown or overexpression ETHE1 and subjected to IP and immunoblotting to examine the effect of ETHE1 on the interaction between GCN2 and eIF2α.

Since ETHE1 is not a putative protein kinase or phosphatase, it cannot directly induce eIF2α phosphorylation. Previous studies have shown that four phosphokinases—GCN2, PERK, PKR, and HRI—can phosphorylate eIF2α [24]. Analysis of ETHE1-interacting proteins detected by LC-MS/MS (Figure 3) found that GCN2 is a potential binding partner of ETHE1 (Figure 5A). Thus, GCN2 was selected for further verification. IP and immunoblotting analyses showed that both eIF2α and ETHE1 interacted with GCN2 (Figure 5B,C). Given that ETHE1 interacts with eIF2α (Figure 4), we hypothesized that ETHE1, eIF2α, and GCN2 could form a ternary complex. This notion was verified using IP and immunoblotting assays (Figure 5D). We then knocked down GCN2 in ETHE1-overexpressing cells using shRNA, and immunoblotting showed that ectopic expression of ETHE1 led to an increase in phosphorylated eIF2α. However, these effects were reversed when GCN2 was depleted (Figure 5E). Together, these results suggest that ETHE1 enhances eIF2α phosphorylation depending on GCN2.

We next examined whether ETHE1 regulates eIF2α phosphorylation by affecting GCN2 expression levels. The results revealed that neither the overexpression of ETHE1 in Hs578T and MDA-MB-231 cells (Figure 5F) nor the knockdown of ETHE1 in SUM159PT and LM2-4175 cells (Figure 5G) notably affected GCN2 protein levels. Next, we tested whether ETHE1 regulates eIF2α phosphorylation by affecting the binding of GCN2 to eIF2α. We discovered that ETHE1 knockdown significantly attenuated the interaction between eIF2α and GCN2 (Figure 5H). In contrast, ETHE1 overexpression significantly enhanced the interaction between eIF2α and GCN2 (Figure 5I). Taken together, these results suggest that ETHE1 enhances eIF2α phosphorylation by promoting the interaction between GCN2 and eIF2α.

### 2.7. ETHE1 Promotes Lung Metastasis of TNBC Cells through eIF2α Phosphorylation–ATF4 Signaling

**Figure 6:**
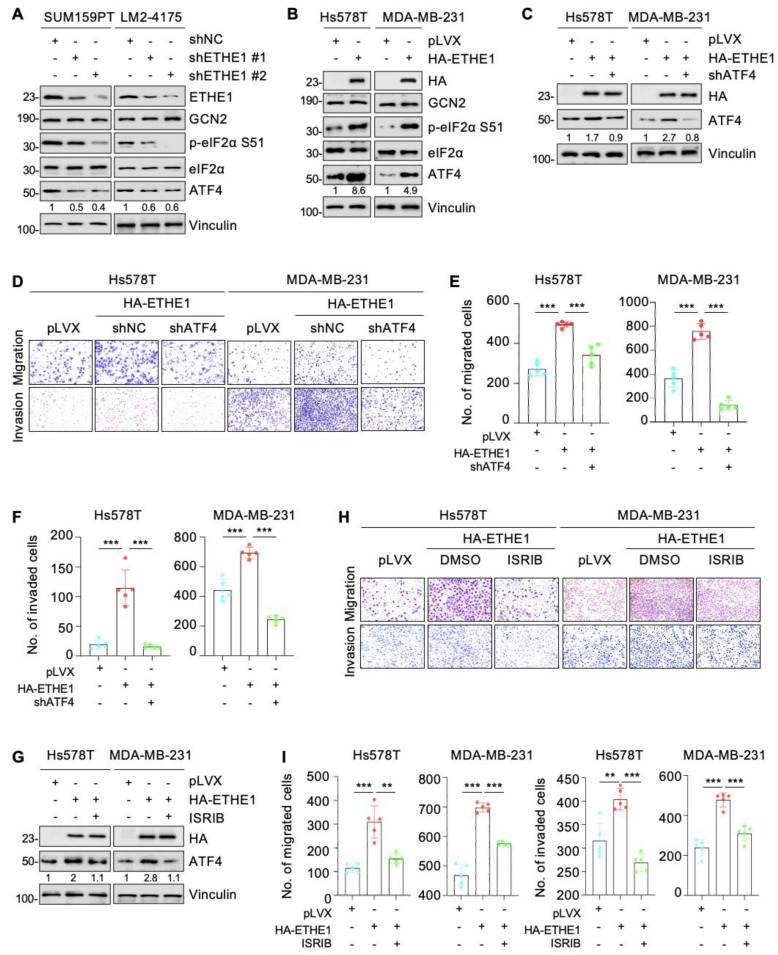
ETHE1 promotes the migration and invasion of TNBC cells in vitro through activating eIF2α phosphorylation–ATF4 signaling. (**A**,**B**) Detection of the expression status of ATF4 in ETHE1 knockdown (**A**) and overexpression (**B**) cells using immunoblotting. (**C**) ETHE1-overexpressing cells were infected with shRNA targeting ATF4 and then detected using immunoblotting. (**D**–**F**) ETHE1-overexpressing cells were infected with shRNA targeting ATF4 and then subjected to Trans-well migration and invasion assays. Typical images (**D**) and quantitative results (**E**,**F**) are shown. (**G**) ETHE1-overexpressing cells were treated with or without ISRIB and then detected using immunoblotting. (**H**,**I**) ETHE1-overexpressing cells were treated with DMSO or ISRIB (200 nM) for 24 h and subjected to Trans-well migration and invasion experiments. Typical images (**H**) and quantitative results (**I**) are shown. ** p < 0.01; *** p < 0.001.

ATF4 is a classical downstream target of eIF2α phosphorylation and plays a vital role in breast cancer cell migration and invasion [25,26,27]. Therefore, we examined whether ETHE1 affects the expression of ATF4. Immunoblotting analysis suggested that depletion of ETHE1 in SUM159PT and LM2-4175 cells downregulated ATF4 protein levels (Figure 6A). Conversely, the overexpression of ETHE1 in TNBC cells upregulated ATF4 protein levels (Figure 6B). To explore whether ETHE1 regulates the migration and invasion of TNBC cells through ATF4, we used shRNA to deplete ATF4 in TNBC cells overexpressing ETHE1 (Figure 6C). Trans-well migration and invasion experiments suggested that ATF4 knockdown compromised the ETHE1-mediated migratory and invasive potentials (Figure 6D–F). ISRIB is an integrated stress response (ISR) inhibitor that potently suppresses eIF2α phosphorylation and its downstream target, ATF4 [28,29]. To further validate the above results, we treated ETHE1-overexpressing cells with ISRIB and demonstrated that ISRIB inhibited ETHE1-induced ATF4 expression (Figure 6G). Trans-well migration and invasion experiments confirmed that ISRIB impaired the ability of ETHE1 to promote migratory and invasive potentials (Figure 6H,I). Collectively, the above results suggest that ETHE1 promotes TNBC cell metastasis by upregulating the expression of ATF4.

**Figure 7:**
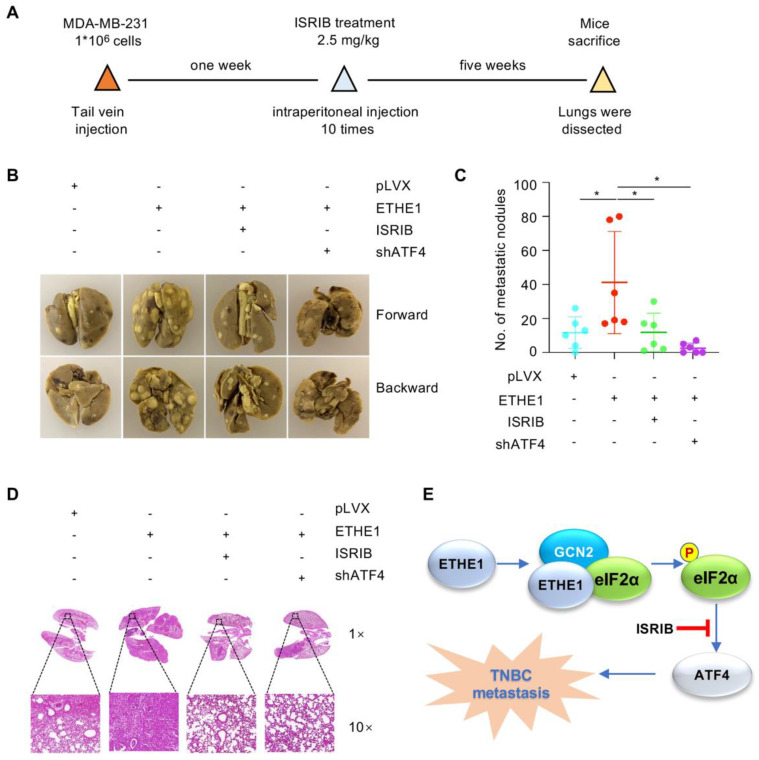
ETHE1 promotes TNBC metastasis in vivo by activating eIF2α phosphorylation–ATF4 signaling. (**A**–**D**) MDA-MB-231 cells (1 × 10^6^) overexpressing pLVX, ETHE1 alone or in combination with shATF4 were injected into female nude mice. After seven days of injection, mice were processed with vector or ISRIB (2.5 mg/kg dissolved in corn oil) through intraperitoneal injection every day for 10 days. The experimental lung metastasis model (**A**), typical images of metastatic lungs (**B**), corresponding quantification results (**C**), and typical images of H&E staining of lung tissues of mice (**D**) are shown. *n* = 6 mice per group, * p < 0.05. (**E**) The proposed working model. ETHE1 was highly expressed in TNBC cells and promoted TNBC metastasis by enhancing GCN2-mediated eIF2α phosphorylation and ATF4 expression. This process can be blocked using the eIF2α phosphorylation inhibitor ISRIB.

Subsequently, to explore whether ETHE1 drives TNBC metastasis by regulating ATF4 in vivo, MDA-MB-231 cells expressing pLVX vector, ETHE1 alone, or ETHE1 with shATF4 were injected into nude mice. After seven days of injection, the mice were processed with or without ISRIB every day for 10 days. Consistent with our in vitro findings, ETHE1 overexpression significantly enhanced lung metastasis of MDA-MB-231 cells (Figure 7A–D). Notably, ISRIB administration and ATF4 depletion significantly impaired the metastatic potential of TNBC cells caused by ectopic expression of ETHE1 (Figure 7A–D). The above results demonstrate that ETHE1 accelerates TNBC metastasis in vivo by regulating ATF4, at least in part, and ISRIB has therapeutic potential in TNBC patients with high ETHE1 expression.

## 3. Discussion

In this study, we reported a previously unknown role of ETHE1 in TNBC metastasis (Figure 7E). Increasing evidence has suggested that ETHE1 is involved in the metabolism of cellular hydrogen sulfide, and mutations in ETHE1 can lead to dysregulation of the physiological process, resulting in ethylmalonic encephalopathy [13,19]. Although ETHE1 plays a key role in ethylmalonic encephalopathy, its role in human cancer is poorly understood. To date, only a few studies have indicated the role of ETHE1 in tumorigenesis of colorectal cancer [14,15]. In this study, we demonstrated that ETHE1 was upregulated in TNBC. Strikingly, ETHE1 had no effect on TNBC cell proliferation and xenograft tumor growth, but it promoted the migration and invasion of TNBC cells (Figure 1, Figure 2, Figure 7 and Figure S1). As ETHE1 mutations inhibit its disulfide dioxygenase activity, we also examined whether the pro-metastatic role of ETHE1 in TNBC was dependent on its enzymatic activity. Hence, we constructed four enzymatically inactive ETHE1 mutants—T152I [19], R163W [20,30], D196N [19], and C247S [22]—for functional experiments. These enzymatically inactive mutations did not affect the ability of ETHE1 to promote TNBC cell migration compared with their wild-type counterpart (Figure S2). Therefore, we believe that the role of ETHE1 in promoting TNBC is independent of its enzymatic activity.

eIF2α plays two pivotal roles in cells. One is to form ternary complexes with initiator tRNA and GTP in the early steps of protein synthesis, and the second is to act as a key component of ISR, which is required to adapt to various pressures [31,32]. eIF2α plays a crucial role in tumor invasion and metastasis [33,34] and its phosphorylation is indispensable for cell migration [23]. In this study, proteomic analysis, immunoprecipitation assays, and immunofluorescence staining revealed that ETHE1 interacts with eIF2α and GCN2 (Figure 3, Figure 4 and Figure 5). Through exploring the regulatory mechanism of ETHE1 on eIF2α, we found that ETHE1 promotes the phosphorylation of eIF2α by recruiting kinase GCN2, thereby regulating the expression of its downstream target, ATF4. Moreover, we showed that eIF2α phosphorylation–ATF4 axis is involved in ETHE1-mediated migration, invasion, and metastasis of TNBC cells both in vitro and in vivo (Figure 6 and Figure 7). Thus, whether the eIF2α phosphorylation inhibitor ISRIB can be used as a clinical inhibitor in TNBC patients with high eIF2α expression needs to be explored further.

Additionally, we found that ETHE1 was upregulated in TNBC, although its upstream regulatory signals remain unclear. Based on the genomic information for ETHE1 in breast cancer, we found that ETHE1 copy number was significantly elevated and closely correlated with its mRNA levels. Therefore, we hypothesized that variations in the copy number of ETHE1 lead to its high expression in breast cancer tissues and boost TNBC metastasis.

## 4. Materials and Methods

### 4.1. Cell Culture and Chemicals

The human embryonic kidney cell line (HEK293T (RRID: CVCL_0063)) and human TNBC cell lines (MDA-MB-231 (RRID: CVCL_0062), HCC1937 (RRID: CVCL_0290), Hs578T (RRID: CVCL_0332), BT549 (RRID: CVCL_1092), HCC1806 (RRID: CVCL_1258), LM2-4175 (RRID: CVCL_5998), and BT20 (RRID: CVCL_0178)) were acquired from the Chinese Academy of Sciences in Shanghai. The SUM159PT cell line (RRID: CVCL_5423) was provided by Suling Liu (Fudan University, Shanghai, China). The cell lines were cultured in DMEM (#L110, BasalMedia, Shanghai, China) containing 10% fetal bovine serum (#10270-106, Gibco, California, America) and 1% penicillin–streptomycin (#S110B, BasalMedia, Shanghai, China). ISRIB was purchased from Selleck Chemicals (#S0706, Houston, America). All cell lines used in this study have been authenticated using short tandem repeat (STR) profiling within the last 3 years. All assays were performed using mycoplasma-free cells.

### 4.2. Clinical Samples and Data

A total of 12 TNBC samples and paired normal breast tissues were obtained from patients who underwent surgery at Fudan University Shanghai Cancer Center (FUSCC). Informed consent was obtained from patients before the operation. The transcriptomic datasets [17] (TNBC tissues: *n* = 360; normal tissues: *n* = 88) and the quantitative proteomic dataset [18] (TNBC tissues: *n* = 90; normal tissues: *n* = 72) were obtained from FUSCC.

### 4.3. Expression Vectors

ETHE1 and eIF2α cDNAs were identified and ligated into pLVX-IRES-NEO or pCDH-CMV-MCS-EF1-Puro vectors to obtain pLVX-HA-ETHE1, pCDH-Flag-ETHE1, pLVX-HA-eIF2α, and pCDH-Flag-eIF2α expression vectors. Point mutations were generated in ETHE1 via PCR-based mutagenesis. The sequences of shRNA targeting ETHE1, ATF4, and GCN2 were obtained from BLOCK-iT™ RNAi Designer (http://rnaidesigner.thermofisher.com/ (accessed on 30 September 2020)) and then subcloned into pLKO.1-TRC vector. Information on DNA constructs, primers, and shRNA targeting sequences is provided in Supplementary Tables S1–S3.

### 4.4. Plasmid Transfection and Lentiviral Infection

Plasmids were transfected into HEK293T cells using a DNA transfection reagent (#TF201201, Tengyi Biotech, Shanghai, China). After 48 h, the viral supernatants were collected and used to infect cells with polybrene (#H9268, Sigma-Aldrich, Missouri, MO, America). After two days, puromycin (#A610593-0025, Sangon, Shanghai, China) or G418 sulfate (#A600958-0005, Sangon, Shanghai, China) was used to screen for the desired cell lines.

### 4.5. Antibodies, Immunoblotting, and Immunoprecipitation (IP)

For immunoblotting, RIPA buffer supplemented with phosphatase and protease inhibitors (#B15003 and #B14002, Bimake, Texas, America) was used to lyse cells and tissues. After quantification using a bicinchoninic acid kit (#20201ES90, Yeasen, Shanghai, China), equal quantities of proteins were separated and then transferred to PVDF membranes (#IPVH00010, Millipore, Bedford, MA, USA). After incubation with antibodies, the protein signals were detected using electrochemiluminescence reagents (#36208ES76, Yeasen, Shanghai, China). For IP assays, cellular extracts were incubated with anti-HA (#B26202, Bimake, Texas, America) or anti-Flag beads (#B26102, Biotool, Beijing, China) at 4 °C, followed by immunoblotting. The information on antibodies is listed in Supplementary Table S4.

### 4.6. Immunofluorescence

The adherent cells were fixed with 4% paraformaldehyde and then blocked with 5–10% goat serum or BSA. Subsequently, cells were incubated with specific primary and secondary antibodies that were conjugated with Alexa Fluor 555 (#4413S, CST, Massachusetts, America) or Alexa Fluor 488 (#4408S, CST, Massachusetts, America). Following this, a DAPI-containing fluor shield mounting medium (#ab104139, Abcam, Cambridge, UK) was used to stain DNA. A Leica fluorescence confocal microscope was used to capture images.

### 4.7. Cell Viability and Colony Formation Assays

A total of 1000 cells per well were seeded into 96-well plates and cell viability was detected on alternate days using the Cell Counting Kit-8 (#40203ES92, Yeasen, Shanghai, China). For colony formation assays, 1000 cells with greater viability were plated into each well of 6-well plates and the cells were cultured for 10–14 days. After fixation using methanol, colonies were stained with 0.5% crystal violet and then counted.

### 4.8. Cell Migration and Invasion Assays

Cell migration or invasion chambers were placed in 24-well plates. A total of 1 × 10^4^–3 × 10^4^ cells were plated in the top chamber. After 16–20 h of incubation, cells in the top side of the chambers were wiped off and cells in the bottom chambers were fixed using 100% methanol, stained with crystal violet, and then counted.

### 4.9. Proteomic Analysis

Total samples were lysed using NP-40 buffer. After quantification, all proteins were subjected to IP experiments and then analyzed using SDS-PAGE. After staining with Coomassie brilliant blue solution, gels were subjected to LC-MS/MS analysis at the mass spectrometry center of Fudan University.

### 4.10. Xenograft Tumors in Nude Mice

For tumorigenic assays, 3 × 10^6^ ETHE1 overexpression or vector MDA-MB-231 cells were injected into female BALB/c nude mice (5-week-old, *n* = 6). After 6 weeks of inoculation, mice were sacrificed via carbon dioxide asphyxiation. The transplanted tumors were weighed and measured. To assess the lung metastatic ability, 1 × 10^6^ ETHE1 overexpression or vector MDA-MB-231 cells were inoculated into the lateral tail vein of mice. After seven days, mice were processed with vector or ISRIB (2.5 mg/kg) via intraperitoneal injection every day for 10 days. After being sacrificed, the lungs from the mice were fixed and the relative numbers of metastatic foci were counted.

### 4.11. Statistical Analysis

The experiments were conducted in triplicate. GraphPad prism 8 was used to analyze the data, and Student’s *t*-test was used to compare differences between the groups. *p* < 0.05 was considered statistically significant.

## Data Availability

The data presented in this study are available within the article text and figures.

## Supplementary Materials

The supporting information can be downloaded at: https://www.mdpi.com/article/10.3390/ijms241914566/s1.

## Abbreviations

**ATF4**, activating transcription factor 4; **eIF2α**, eukaryotic translation initiation factor 2 subunit alpha; **ETHE1**, ethylmalonic encephalopathy protein 1; **GCN2**, general control nonderepressible 2; **ISRIB**, integrated stress response in inhibitor; **TNBC**, triple-negative breast cancer.
