# Supplementary material for: ETHE1 Accelerates Triple-Negative Breast Cancer Metastasis by Activating GCN2/eIF2α/ATF4 Signaling
Source: Int J Mol Sci. 2023 Sep 26;24(19):14566. doi: 10.3390/ijms241914566 (PMC10572406; doi:10.3390/ijms241914566)
Supplement: Supplementary file 1 [file ijms-24-14566-s001.zip › ijms-2529295-supplementary.pdf]

Supplementary information for

ETHE1 accelerates triple-negative breast cancer metastasis by activating GCN2/eIF2α/ATF4 signaling

This Supplementary information contains:

Supplementary Figures: 2

Supplementary Tables: 4

Supplementary figures and figure legends

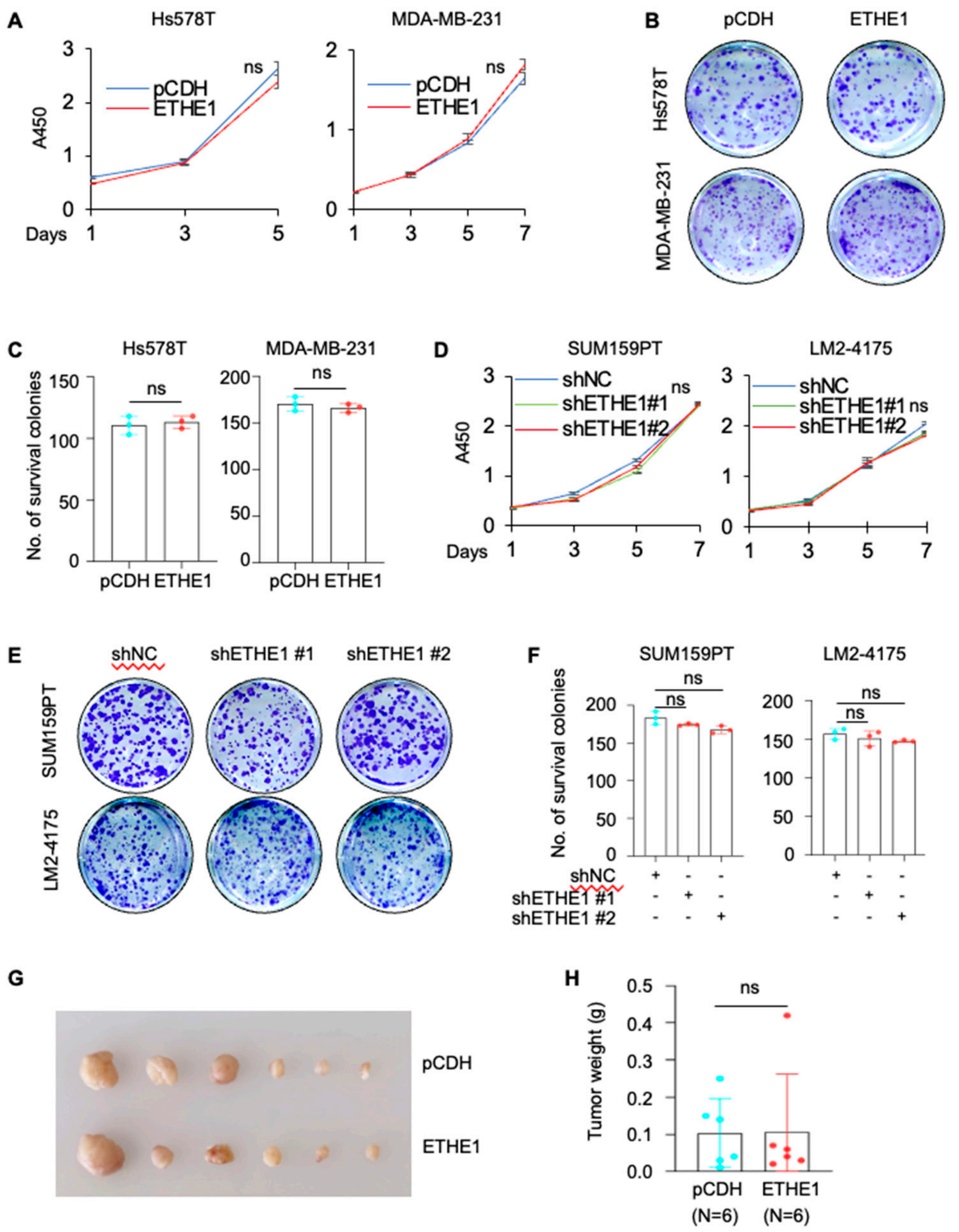

Figure S1. ETHE1 has no remarkable effects on TNBC cell proliferation and xenograft tumor growth

(A-C) Hs578T and MDA-MB-231 cells stably overexpressing ETHE1 were subjected to CCK-8 assays (A) and colony formation assays (B-C). Representative images of survival colonies (B) and corresponding quantitative results (C) are shown. **(D-F)** SUM159PT and LM2-4175 cells stably expressing shNC and shETHE1 (#1 and #2) were subjected to CCK-8 assays (D) and

colony formation assays (E-F). Representative images of survival colonies (E) and corresponding quantitative results (F) are shown. **(G-H)** A total of 3×10^6 MDA-MB-231 cells stably expressing pCDH or Flag-ETHE1 were injected into the mammary fat pad of 5-week-old BALB/c female nude mice (n=6). After 6 weeks of inoculation, mice were sacrificed. The xenograft tumors were weighed and calculated. Representative images of xenograft tumors (G) and tumor weight (H) are shown.

ns, no significance.

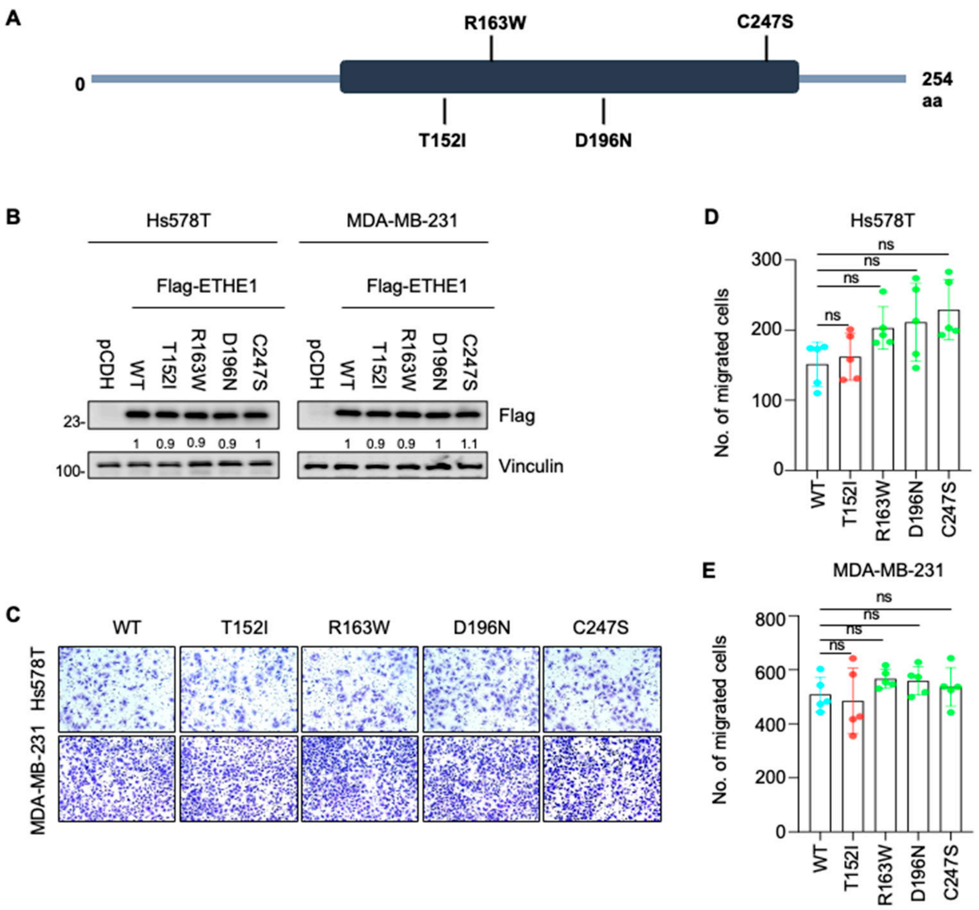

Figure S2. ETHE1 promotes migratory and invasive potential of TNBC cells independent of its enzymatic activity

(A) Common ETHE1 mutations are shown. **(B)** Hs578T and MDA-MB-231 cells stably expressing wild-type and mutant ETHE1 were subjected to immunoblotting analyses with the indicated antibodies. **(C-E)** Hs578T and MDA-MB-231 cells stably expressing wild-type and mutant ETHE1 were subjected to Transwell migration assays. Representative images of migrated cells (C) and quantitative results are shown in D and E.

ns, no significance.

Supplementary Tables

Table S1. Information for the used expression vectors

Plasmids	Sources	Vectors
HA-ETHE1	Subcloned	pLVX-IRES-Neo
HA-EIF2S1	Subcloned	pLVX-IRES-Neo
Flag-ETHE1	Subcloned	pCDH-CMV-MCS-EF1-Puro
Flag-EIF2S1	Subcloned	pCDH-CMV-MCS-EF1-Puro
shETHE1 #1	Subcloned	pLKO.1-TRC
shETHE1 #2	Subcloned	pLKO.1-TRC
shGCN2	Subcloned	pLKO.1-TRC
shATF4	Subcloned	pLKO.1-TRC

Table S2. Primers used for molecular cloning

Plasmids	Primers	Sequences
HA-ETHE1	Forward	GGATCTATTTCCGGTGAATCTAGAGCCACCATGGCGGAGGCTGTACTG
	Reverse	GGATCCGCGGCCGCTCTTTAAGCGTAGTCTGGGACGTCGTATGGGTAGAATTCGGCAGTGGGTGTCTGCAC
Flag-ETHE1	Forward	GATTCTAGAGCTAGCGAATTCGCCACCATGGCGGAGGCTGTACTGAGGGTCG
	Reverse	ATGGTCTTTGTAGTCGGATCCGGCAGTGGGTGTCTGCACCCCACAG
Flag-EIF2S1	Forward	GATTCTAGAGCTAGCGAATTCGCCACCATGCCGGGTCTAAGTTGTAGATTTTAT
	Reverse	ATGGTCTTTGTAGTCGGATCCATCTTCAGCTTTGGCTTCCATTTC
T152I	Forward	CAGCATGGCCTTCATCGGAGATGCCCTGTTG
	Reverse	CAACAGGGCATCTCCGATGAAGGCCATGCTG
R163W	Forward	GTTGATCCGTGGGTGTGGGTGGACAGACTTCCAGCAAG
	Reverse	CTTGCTGGAAGTCTGTCCACCCACACCCACGGATCAAC
D196N	Forward	CTGATCTACCCTGCTCACAACTACCATGGGTTCACAGTG
	Reverse	CACTGTGAACCCATGGTAGTTGTGAGCAGGGTAGATCAG
C247S	Forward	TCCAGCCAACATGCGCAGCGGGGTGCAGACA
	Reverse	CGGTTGTACGCGTCGCCCCACGTCTGTGGGT

Table S3. shRNA targeting sequences

Targets	Primers	Sequences
shETHE1 #1	Forward	CCGGGCCCAGGCTGACTTACACATTCTCGAGAATGTGTAAGTCAGCCTGGGCTTTTTG
	Reverse	AATTCAAAAAGCCCAGGCTGACTTACACATTCTCGAGAATGTGTAAGTCAGCCTGGGC
shETHE1 #2	Forward	CCGGGGCTGACTTACACATTGAGGACTCGAGTCCTCAATGTGTAAGTCAGCCTTTTTG
	Reverse	AATTCAAAAAGGCTGACTTACACATTGAGGACTCGAGTCCTCAATGTGTAAGTCAGCC
shGCN2	Forward	CCGGGCAGAGAAGCTTCCGATAATCCTCGAGGATTATCGGAAGCTTCTCTGCTTTTTG
	Reverse	AATTCAAAAAGCAGAGAAGCTTCCGATAATCCTCGAGGATTATCGGAAGCTTCTCTGC
shATF4	Forward	CCGGGATCCAGTACCTGAAAGATTTCTCGAGAAATCTTTCAGGTACTGGATCTTTTTG
	Reverse	AATTCAAAAAGATCCAGTACCTGAAAGATTTCTCGAGAAATCTTTCAGGTACTGGATC

Table S4. Antibodies used in this study

Name	Company	Catalog Number
GCN2	Abcam	ab134053
p-eIF2α S51	ABclonal	AP0692
ATF4	SinoBiological	310527-T32
ETHE1	SinoBiological	14681-T60
eIF2α	SinoBiological	101112-T38
Flag	Sigma	GNI4110-FG
Vinculin	Sigma	V9131-0.2ML
HA	CST	3724S
Rabbit IgG	CST	7074V
Mouse IgG	CST	7076V
